# CAR-T cell therapy in myeloma: hopes and hurdles

**DOI:** 10.1097/BS9.0000000000000148

**Published:** 2023-01-04

**Authors:** Jean Luc Harousseau

**Affiliations:** Institut de Cancérologie de l’Ouest, Saint Herblain, France

Immune therapy is a new avenue in the treatment of multiple myeloma (MM). The naked anti-38 antibodies daratumumab and isatuximab are already used in frontline therapy after excellent results have been achieved in relapsed/refractory MM (RRMM).

The second step was the development of immune therapies targeting B cell maturation antigen (BCMA). Genetically modified autologous chimeric antigen receptor (CAR)-T cell therapy BCMA-directed were initially tested in heavily pretreated patients with RRMM exposed to the 3 main therapeutic classes, immunomodulatory drugs (iMiDs), proteasome inhibitors, and anti-CD38 antibodies (triple-class exposed). Efficacy results were unprecedented in this context and Idecabtagene vicleucel (ide-cel or Abecma) was the first BCMA-directed CAR-T cell therapy approved in MM by both Federal Drug Administration (FDA) and European Medicines Agency (EMA). This approval was based on the results of the KarMMa Phase 2 study in 128 patients with RRMM who had previously received a median number of 6 lines of therapy (LOT).^1^ The response rate (RR) was 73%, including 33% of complete response (CR) or better and 26% negative minimal residual disease (MRD). However, median progression-free survival (PFS) was only 8.8 months. The Cartitude 1 Phase1b/2 evaluated ciltacabtagene autoleucel (cilta-cel) another CAR-T cell therapy with 2 BCMA-targeting single domain antibodies in 97 heavily pretreated patients with RRMM.^2^ Results were recently updated and with a median follow-up of 27.7 months,^3^ the results were even more impressive with a 98% RR, including 82.5% CR or better, and 92% negative MRD in evaluable patients. The 27-month PFS was 55%. With these results, Cilta-cel (Carvykti) was FDA and EMA approved in 2022.

Both CAR-T cell therapies induced prolonged and recurrent cytopenias and 2 specific adverse events, cytokine release syndrome (CRS) and neurologic complications including immune effector-cell–associated neurotoxicity syndrome (ICANS). These toxicities may be severe and require specific management by skilled health professionals.

Ciltacel appears the most promising CAR-T cell therapy in RRMM, although direct comparison of the KarMMa and Cartitude-1 trials should be cautious since the populations treated were not fully identical. It should be noted that an identical construct called LCAR-B38M was previously evaluated in China, with 2 investigator-initiated Phase 1 studies which both also yielded excellent RR of 88% and 75% CR or better. Mi et al^4^ just published the results of a Phase 2 open-label trial of cilta-cel at the target dose of 0.75 × 10^6^ in 48 Chinese patients with RRMM who had previously received a median number of 4 LOT. The objective was to confirm results of Cartitude-1 study in an ethnically different population, in the context of medical practice and standard of care in China and with manufacturing in China. The patients treated was actually slightly different from patients included in the Cartitude-1 trial, notably there were less triple-class refractory patients (only 19% vs 88%), since Daratumumab is not frequently used in China, but more patients with high risk cytogenetics. Results were almost as good with 89.6% RR including 77.1% ≥CR. The 18 months PFS was 66.8%. Therefore, this trial confirms the outstanding efficacy of a single shot infusion of cilta-cel in heavily pretreated patients in Chinese patients, which of special importance in a country where many new agents are not yet available.

However 2 concerns should be underlined. First, the incidence of grade 3/4 CRS was high (35.4%) and 10 patients died from complications including infection and hemorrhage. This confirms that CAR-T cell therapy should be administered only in specialized/authorized units with adequate resources and adapted prophylaxis and treatments of potential complications. Second, 16 of the 64 patients who underwent apheresis could not receive the CAR-T infusion due to the delay between apheresis and cilta-cel infusion (5–7 weeks). This is an usual concern with CAR-T cell therapy when recruited patients have very advanced disease. Since the delay may sometimes be as long as 10 weeks, bridging therapy may not be effective enough to stop a rapid disease progression, and the time needed for a personalized procedure is an argument in favor of using CAR-T at earlier stages.

Two other BCMA-directed immune therapies can be used in case of CAR-T cell unavailability or of rapid progression since they are produced off the shelf and can be administered immediately if available: antibody-drug conjugates (ADC) and mostly bispecific T cell engagers antibodies (BSA). The Dreamm 2 Phase 2 trial of the first-in-class ADC Belantamaf Mafodotin showed encouraging results with approximately 30% RR in patients who previously received a median of 7 LOT (and ≥4 in 84% of patients).^5^ Some responses were durable with a median duration of response of 11 months. The most relevant adverse event was keratopathy.

Bispecific antibodies targeting both BCMA and CD3 T cells are another attractive alternative. The MAJESTEC-1 Phase ½ trial of Teclistamab subcutaneously once per week in 165 heavily pretreated patients (median 5 prior LOT, 78% triple-class refractory) yielded 63% RR with 39.4% ≥CR.^6^ The median duration of response was 18.4 months. CRS and neurologic toxicity were described (72% and 24%, respectively) but were mostly grade 1–2. Infections were the most important concern. Based on these results, teclistamab was recently approved by EMA and FDA. Other BCMA-directed bispecific antibodies are currently developed (erlanatamab, AMG-701, ABBV-383, REGN-5458).^7^

Since BCMA loss or downregulation is one of the possible causes of anti-BCMA therapy failure,^8^ one can ask whether CAR-T cells are still active after failure of an BCMA-targeting antibody (ADC or BSA). Preliminary results with cilta-cel are encouraging with approximately 60% of responses in the 20 patients who could receive the CAR-T cells.^9^ As failures or relapses can occur after both types of BCMA-directed immune therapies (CAR-T cells or antibodies), other surface membrane antigens of interest are being evaluated: G-protein coupled receptor family C group 5 member D (GPRC5D) or Fc receptor homologue 5 (FCRH5). Preliminary results with Talquetamab (anti-GPRC5D BSA) or Cevostamab (FCRH5 BSA) or with MCARH109 (GPR5CD CAR-T cells) are encouraging.^10^

There is currently a very active clinical research on CAR-T cells in MM with more than 170 trials registered on clinicaltrials.gov including more than 80 in China. These trials aim first at improving CAR-T cell constructs to reduce resistance and improve availability, but also to evaluate approved CAR-T cells in earlier relapses and even in frontline treatment. But at the same time, in many countries access to CAR-T cell therapy remains limited because of cost and manufacturing issues.
